# Vitamin D Receptor Gene Polymorphism ApaI as a Predisposing Factor for Psoriasis and Its Relation With Serum Vitamin D Levels and Psoriasis Severity

**DOI:** 10.7759/cureus.32715

**Published:** 2022-12-19

**Authors:** Ghadah Alhetheli, Mohammed S Al-Dhubaibi, Saleh S Bahaj, Ahmed I AbdElneam

**Affiliations:** 1 Department of Dermatology, College of Medicine, Qassim University, Buraydah, SAU; 2 Departments of Dermatology, Shaqra University, Dawadmi, SAU; 3 Department of Microbiology and Immunology, Sana'a University, Sana'a, YEM; 4 Department of Biochemistry, College of Medicine, Shaqra University, Shaqra, SAU; 5 Molecular Genetics and Enzymology Department, Human Genetics and Genome Research Institute, National Research Center, Cairo, EGY

**Keywords:** apai gene polymorphism, psoriasis, rs7975232 polymorphism, vdr polymorphisms, vitamin d receptor gene polymorphisms

## Abstract

Background and Aim: Psoriasis is a chronic, relapsing and inflammatory multisystemic disease with both genetic predisposition and autoimmune pathogenic traits. Several types of vitamin D receptor (VDR) polymorphisms have been investigated as a predisposing factor for psoriasis susceptibility with controversial results. However, the exact pathophysiological effect of the VDR gene on psoriasis susceptibility remains poorly understood. We aimed to determine whether VDR gene polymorphisms, specifically rs7975232 (ApaI), afford psoriasis susceptibility in a given community in Saudi Arabia. Also, to assess its possible relation with disease severity.

Subjects and Methods: In a comparative case-control study comprising 53 psoriatic patients and 41 matched healthy controls, we measured serum ApaI levels, and the PCR-RFLEP technique detected ApaI genetic polymorphism (rs7975232) for both groups. Serum vitamin D level was measured in both groups.

Result: Our results revealed that A/A genotype of ApaI was significantly more predominant in patients than controls, while A/a genotype was more common in healthy subjects. Furthermore, A allele was significantly over-represented in the patients' group compared to the controls (P≤0.001). Serum vitamin D levels were significantly higher in mild psoriatic patients than in those with moderate and severe types (P=0.002). Mild psoriatic patients with a/a genotypes have higher vitamin D levels than severe patients with A/A genotypes and A/a moderate patients (P≤0.001).

Conclusion: Our data indicated clearly that VDR gene polymorphism, namely ApaI, is associated with psoriasis susceptibility. Furthermore, serum vitamin D level in psoriatic patients varies among different ApaI genotypes, where it is lowest in AA genotype.

## Introduction

Psoriasis is a chronic, relapsing, inflammatory, and multisystemic disease that predominantly affects the skin and joints. It has complex genetic and environmental backgrounds [1]. The reported prevalence of psoriasis ranges between 0.09% and 11.43%, with at least 100 million diseased individuals worldwide, making psoriasis a serious global problem [2].

Psoriasis symptoms can affect both skin and nails with variable severities. About a quarter of patients suffer from moderate to severe diseases. Characteristic cutaneous manifestations are scaly, well-defined, erythematous papules and plaques that are often pruritic or painful, causing impairments in quality of life (QOL) [3].

The superfamily of steroid, retinoid, and thyroid hormone receptors includes the ligand-activated transcription factor known as vitamin D receptor (VDR). In various tissues, it mediates the genomic effects of 1,25-dihydroxy vitamin D. At the skin level, 1,25-dihydroxy vitamin D3 [1,25(OH)2D3] is a ligand of VDR, forming a receptor-hormone complex that binds with specific hormone response elements of a target gene. This leads to the inhibition of the proliferation of cultured human keratinocytes and initiation of terminal differentiation of these cells, in addition to the modulation of immune systems in different ways [4]. 

VDR gene variants have been linked to a variety of disorders, including psoriasis, systemic lupus erythematosus, breast cancer, prostate cancer, malignant melanoma, osteoarthritis, diabetes, primary hyperparathyroidism, and atherosclerotic coronary artery disease [5-7].

The VDR gene in chromosome 12q13.11 has more than 200 single nucleotide polymorphisms (SNPs). The most investigated types of VDR gene polymorphisms in psoriasis are rs11568820 (Cdx2), rs2228570 (FokI), rs731236 (TaqI), rs7975232 (ApaI), rs1544410 (BsmI), rs757343 (Tru9I), and rs4516035 (EcoRV). As with many association studies, the results are highly controversial and non-conclusive. The exact VDR function remains not well understood. Several studies have found an association between VDR gene polymorphisms and response to psoriasis treatment [4,8,9].

To our knowledge, there has not been researching assessing VDR gene polymorphism rs7975232 (ApaI) as a risk factor for developing psoriasis in Saudi Arabia. Therefore, we aimed in this study to determine whether the VDR gene polymorphisms, specifically rs7975232 (ApaI), confer psoriasis susceptibility in a given community in Saudi Arabia. Additionally, to determine the influence of ApaI genotype on changing serum 25-hydroxyvitamin D (25(OH)D) concentration in psoriasis patients and its relation with psoriasis severity.

## Materials and methods

Subjects

In this comparative case-control study, we randomly recruited 53 patients (24 males and 29 females) at the Dermatology Outpatient Department of Shaqra University, Saudi Arabia, from January 2021 until July 2021. It was carried out in accordance with the Declaration of Helsinki's rules and Approved by the Medical Research Ethics Committee with project number CMD/DWD/SU/2021/01/031. Before recruitment and following a sufficient and simple explanation of the study's purpose and nature, all individuals provided written informed consent.

The following are the inclusion criteria of our study; we enrolled adult male and female patients of any age who had a clinically established diagnosis of psoriasis but did not have any systemic involvement. They are off any systemic, topical, or phototherapy treatment for the last eight weeks. We excluded patients on current systemic treatment for psoriasis, pregnancy, lactation, malignancies, active liver disease, renal disorders, infections, and alcohol use.

Patients were classified according to body surface area (BSA) into severe psoriasis vulgaris greater than 10% of the body surface, moderate psoriasis vulgaris 5%-10% of the body surface, and mild psoriasis vulgaris less than 5% of the body surface [10].

The healthy control group was composed of 41 age and sex-matched healthy subjects (22 males and 19 females); they had no clinical evidence or family history of psoriasis or other autoimmune disorder. Both groups had undergone complete physical and clinical examinations and biochemical tests and were genetically unrelated. Both groups were off any kind of vitamin D supplement for the past 12 weeks.

Genomic DNA extraction

Blood samples were collected on Na2EDTA as an anticoagulant. According to manufacturer instructions for blood protocol, genetic DNA was purified from 200μl of whole blood with the QIAamp® DNA BloodMini Kit (Qiagen, Hilden, Germany).

Detection of ApaI polymorphism using PCR-RFLP

To determine the genotyping of ApaI polymorphism, a genomic DNA fragment was amplified by using the Polymerase Chain Reaction (PCR) method with a pair of oligonucleotide primers: The upstream primer sequence was: 5' CAACCAAGACTACAAGTACCGCGTCAGTGA -3'; and the downstream primer was: 5'- CACTTCGAGCACAAGGGGCGTTAGC-3'. The primers were blasted to the gene bank database https://blast.ncbi.nlm.nih.gov/Blast.cgi. The PCR cycle conditions were denaturation at 94 oC for 4 min, followed by 35 cycles at 94 oC for 30 s, 65 oC for 30 s, 72 oC for 2 min, and one final extension cycle at 72 oC for 4 min using 100-200ng of genomic DNA. The 2000-bp PCR product that had been amplified was digested with the ApaI restriction enzyme (Gibco BRL). In a 10 l reaction with the manufacturer's buffer and 5 units of ApaI restriction enzyme, the PCR product in 5 l was digested for three hours at 37 oC. The product that had been digested by the ApaI enzyme was electrophoretically run at 100 volts for 30 minutes on a 3% agarose gel containing 0.5% g/ml ethidium bromide. Common allele A (wild-type allele) was designated as the absence of the ApaI restriction site (2000 bp), while infrequent allele a (mutant allele) was designated as the presence of the restriction site (1700 bp and 300 bp fragments). Genotypes were assigned as homozygotes for the common allele (AA) and homozygotes for the infrequent allele (aa). The presence of 2000, 1700, and 300 bp fragments were assigned as heterozygotes (Aa) [11].

Measurement of serum vitamin D concentration

Serum 25-hydroxyvitamin D (25(OH)D) concentrations were assessed in the patients at baseline. Vein blood samples were taken, and the Roche Cobas e411 was used to analyze them within 24 hours (Roche Diagnostics System, Switzerland). 25(OH)D blood levels were divided into acceptable (>20 ng/ml), inadequate (12-20 ng/ml), and deficient (12 ng/ml) categories based on the recommendations of the Food and Nutrition Board of the Institute of Medicine [12].

Statistical analysis

Recorded data were analyzed using the IBM Corp. Released 2011. IBM SPSS Statistics for Windows, Version 20.0. Armonk, NY: IBM Corp. To examine the mean and percentage differences, an analysis of variance (ANOVA) test was conducted between more than two means, t-tests, and chi-squares. P ≥ 0.05 was regarded as insignificant, whereas a significance level of P ≤ 0.001 was regarded as highly significant. The qualitative data were expressed as frequency and percentage, whilst the quantitative data were expressed as a mean ± and standard deviation (SD).

## Results

**Figure 1:**
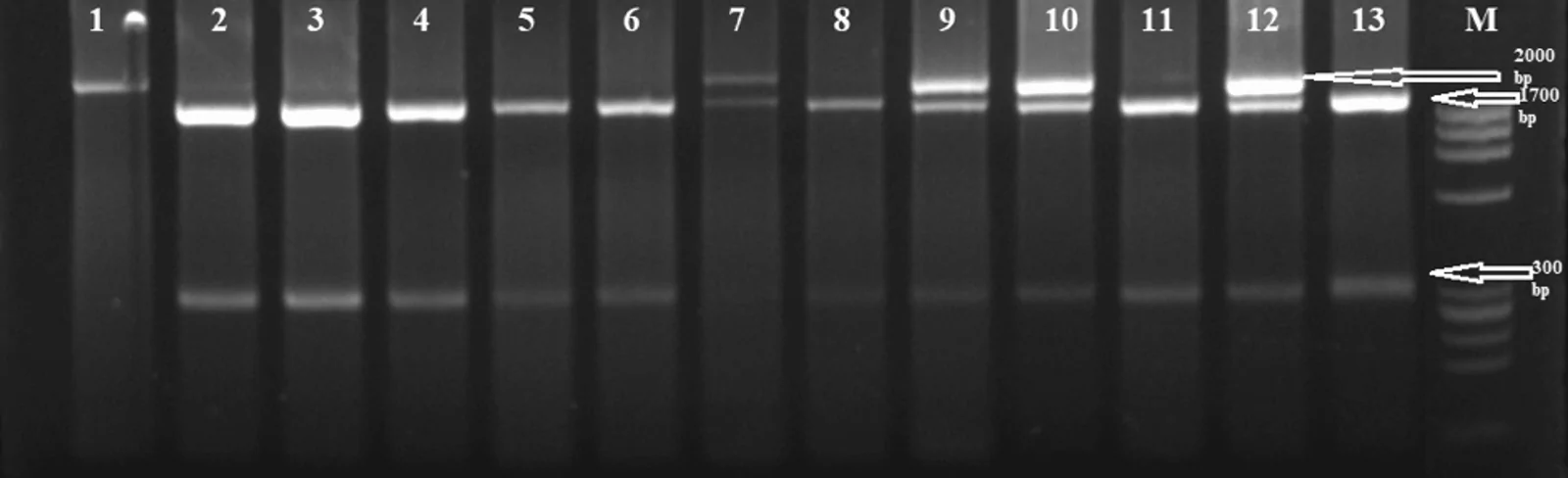
Restriction enzyme analysis for ApaI gene polymorphism. Lane 1 with 2000 bp (AA genotype). Lane 2, 3, 4, 5, 6, 8, 11, and 13 with 1700 bp and 300 bp (aa genotype). Lane 7, 9, 10, and 12 with 2000 bp, 1700 bp, and 300 bp (Aa genotypes). Lane M phi x 174 marker.

Clinical and demographic data of both patients and control groups are shown in Table 1. We amplified specific segments of ApaI gene polymorphism by PCR and then digested it with ApaI restriction enzyme (Figure 1).

**Table 1:** General and laboratory characteristics of psoriatic patients and healthy control. SD: Standard deviation; N: Number, **Mild significant differences P ≤ 0.005; %: percentage; NA: Not applied.

Parameters	Patients N (53) Mean ± SD	Control N (41) Mean ± SD	P-value
Age	45.3 ± 5.4	41.5 ± 6.7	0.421
Male (N, %)	24 (45.3%)	22 (53.7%)	0.485
Female (N, %)	29 (54.7%)	19 (46.3%)	0.765
Severe psoriasis vulgaris (N, %)	16 (30.2%)	--------	NA
Moderate psoriasis vulgaris (N, %)	18 (34%)	--------	NA
Mild psoriasis vulgaris (N, %)	19 (35.8%	--------	NA
Presence of family history (N, %)	33 (62.3%)	--------	NA
Absent of family history (N, %)	20 (37.7%)	--------	NA
Serum vitamin D level (ng/ml)	22.5 ± 4.9	38.9 ± 5.4	0.002 **

A comparison of ApaI gene polymorphism genotypes between patients and controls groups showed that in the psoriasis group, the A/A genotype was the most common (43.4%) with a statistically significant difference, followed by A/a (34%) and then a/a (22.6%). On the other hand, the A/a genotype was significantly predominant in the control group (49%), followed by a/a and A/A genotypes with 34% and 17%, respectively (Table 2).

**Table 2:** Distribution of VDR ApaI gene genotypes among psoriatic patients and healthy control. N: Number; *: High significant differences (P≤0.001), %: Percentage

Genotypes	Patients (N, %)	Controls (N, %)	P-Value
A/A	23 (43.4%)*	7 (17%)	≤0.001 *
A/a	18 (34%)	20 (49%)*	≤0.001 *
a/a	12 (22.6%)	14 (34%)	0.899

Our results revealed that the A allele was over-represented considerably in the patient's group compared to the controls group (60% vs. 41.5%) (P ≤0.001). At the same time, there is no significant variation between patients and controls in (a) allele (P=0.426) (Table 3).

**Table 3:** Comparison of allele frequency in psoriatic patients and healthy control. N: Number; *: High significant differences (P≤0.001), %: Percentage

Allele frequency	Patients (N, %)	Controls (N, %)	P-Value
A	64 (60%)	34 (41.5%)	≤0.001 *
a	42 (40%)	48 (58.5%)	0.426

Patients with a/a genotypes have statistically significantly higher serum vitamin D (P =0.005) compared to other genotypes A/a and A/A Table 4.

**Table 4:** Comparison of serum vitamin D level and ApaI gene genotypes in psoriatic patients. SD: standard deviation, N: number; *: Mild significant differences (P≤ 0.005).

Parameters	A/ A (N= 23)	A/a (N=18)	a/a (N = 12)	P-Value
Serum vitamin D level (ng/ml) (mean ± SD)	25.4 ± 6.2	32.4± 5.98	41.8 ± 4.88.*	0.005 *

Comparing serum vitamin D levels among clinical types of psoriasis, our results showed that serum vitamin D level was significantly higher in mild psoriasis vulgaris patients than in those with moderate psoriasis and severe psoriasis vulgaris (P=0.002) (Table 5).

**Table 5:** Comparison of serum vitamin D level and variable clinical types of psoriatic patients. SD: Standard deviation, N: Number; **: Mild significant differences (P≤ 0.005).

Parameters	Severe psoriasis vulgaris (N = 16)	Moderate psoriasis vulgaris (N = 18)	Mild psoriasis vulgaris (N = 19)	P-Value
Serum vitamin D level (ng/ml) (mean ± SD)	19.8 ± 4.77	28.7± 3.89	34.2 ± 5.92	0.002 **

Out of 16 patients with severe psoriasis vulgaris, 10 patients have A/A genotypes, while in moderate psoriasis vulgaris, 15 out of 18 have A/a genotypes. On the other side, out of 19 patients with mild psoriasis, only nine patients have a/a genotypes. When measuring those patients' serum vitamin D levels, it was statistically significantly higher in mild psoriasis vulgaris patients than in severe and moderate psoriasis vulgaris patients (P≤0.001) (Table 6).

**Table 6:** Comparison of serum vitamin D level and variable clinical types of psoriasis according to ApaI genotypes. SD: Standard deviation, N: Number; *: High significant differences (P≤0.001).

Parameters	Severe psoriasis vulgaris (N = 10) have A/A genotypes	Moderate psoriasis vulgaris (N = 15) have A/a genotypes	Mild psoriasis vulgaris (N = 9) have a/a genotypes	P-Value
Serum vitamin D level (ng/ml) (mean±SD)	15.99 ± 5.32	24.8± 6.51	34.39± 4.15	≤0.001 *

## Discussion

Studies on VDR gene polymorphisms and allelic frequencies have been conducted on different populations; however, the results are controversial [6]. Our study identified an association between the VDR gene polymorphisms, namely ApaI, and psoriasis in a given community of Saudi patients with psoriasis. These findings suggest that VDR gene polymorphisms may play an important role in the etiopathogenesis of psoriasis.

The VDR gene is located on 12q12-14. The three mostly studied polymorphisms in psoriasis are, BsmI, ApaI (both in intron 8), and TaqI (in exon 9). All have been identified at the 30 untranslated regions (UTR) of the gene and, importantly, shown to be in strong linkage disequilibrium (LD).

In our study, we found that the AA genotype was significantly more prevalent in psoriasis patients than controls, and this is in accordance with the findings of a study done by Park et al. 1999 on Korean patients who reported significant differences in VDR genotype and allele frequencies (ApaI allele A) between psoriasis patients and normal controls, especially at early onset psoriasis. They suggested that disequilibrium in VDR gene expression or allelic frequencies could be a risk factor for the development of psoriasis [13]. Similar findings were found by Zhou et al. 2014 who identified an association between one of the ApaI VDR gene polymorphisms and psoriasis in a Han population in northeastern China [14]. Also, another study on the Turkish population reinforced this association between ApaI polymorphism and psoriasis [15].

On the other hand, other studies found no associations between VDR ApaI polymorphism and psoriasis compared with healthy controls [7,15-19].

Interestingly ApaI polymorphisms are in the intronic site of the gene. Their importance may be explained by introns playing a role in controlling expression through RNA editing and alternative splicing [20].

In the present study, serum vitamin D level was found to be significantly lower in the psoriasis group than in controls, and this is similar to the results of other studies, and the reason is that the psoriatic patients are less likely to expose their skin to sunlight [19,21,22].

Interestingly, in comparison of serum vitamin D levels in variable clinical types of psoriasis according to ApaI genotypes, we found that those who carry A/A genotype from patients with severe psoriasis have highly significantly lower serum levels of vitamin D than those who carry A/a genotype from patients with moderate psoriasis and those who carry the a/a genotype from patients with mild psoriasis.

Limitation

It is necessary to conduct additional clinical research with a large sample size to look into the molecular mechanism behind the link between the ApaI gene polymorphism and psoriasis. Studying different haplotypes rather than a single VDR gene polymorphism could be more helpful in understanding the pathophysiological mechanism of the VDR gene in the development of psoriasis.

## Conclusions

In conclusion, to our knowledge, this is the first study of VDR gene polymorphism as a predisposing factor for developing psoriasis in Saudi Arabia. We found that the ApaI AA genotype is significantly more prevalent in psoriatic patients than in healthy control and that the AA genotype is also associated with lower serum vitamin D levels.
