# Correction: Vitamin D Receptor Gene Polymorphism ApaI as a Predisposing Factor for Psoriasis and Its Relation With Serum Vitamin D Levels and Psoriasis Severity

**DOI:** 10.7759/cureus.c503

**Published:** 2026-11-12

**Authors:** Ghadah Alhetheli, Mohammed S Al-Dhubaibi, Saleh S Bahaj, Ahmed I AbdElneam

**Affiliations:** 1 Department of Dermatology, College of Medicine, Qassim University, Buraydah, SAU; 2 Departments of Dermatology, Shaqra University, Dawadmi, SAU; 3 Department of Microbiology and Immunology, Sana'a University, Sana'a, YEM; 4 Department of Biochemistry, College of Medicine, Shaqra University, Shaqra, SAU; 5 Molecular Genetics and Enzymology Department, Human Genetics and Genome Research Institute, National Research Center, Cairo, EGY

This article has been corrected to change Dr. Ghadah Alhetheli’s affiliation to “Department of Dermatology, College of Medicine, Qassim University, Buraydah, SAU.”

